# Overview of Proteomic Analysis of Amyloid Plaques and Neurofibrillary Tangles in Alzheimer’s Disease

**DOI:** 10.3390/biom15091310

**Published:** 2025-09-11

**Authors:** Amber Grewal, Simran Raikundalia, Joseph Zaia, Manveen K. Sethi

**Affiliations:** 1 Center for Biomedical Mass Spectrometry, Department of Biochemistry and Cell Biology, Boston University Chobanian and Avedisian School of Medicine, Boston, MA 02118, USA; amgrewa@bu.edu (A.G.); simranr@bu.edu (S.R.); jzaia@bu.edu (J.Z.); 2 Bioinformatics Program, Boston University, Boston, MA 02215, USA

**Keywords:** Alzheimer’s disease, amyloid plaques, neurofibrillary tangles, laser microdissection, mass spectrometry

## Abstract

In this review, we describe the methods used for the extraction and mass spectrometry proteomics analysis of amyloid plaques and neurofibrillary tangles (NFTs), the two primary pathological hallmarks of Alzheimer’s disease (AD). We also provide a comprehensive overview of the mass spectrometry-based studies conducted to analyze these pathological features. AD is the most prevalent form of dementia and the sixth leading cause of death in the United States. While the current treatments can alleviate early-stage memory and cognitive symptoms, they do not offer a cure. Thus, there is a pressing need to deepen our understanding of the neuropathological mechanisms underlying AD and to develop more effective therapeutics. In-depth mass spectrometry-based proteomics analyses of AD pathology—specifically, extracellular the Aβ plaques found in extracellular spaces and blood vessel walls and intraneuronal NFTs composed of the microtubule-associated protein tau—may offer molecular-level observations that contribute to the understanding of the biological context of plaque and NFT formation and support the discovery of potential biomarkers and therapeutic targets for AD.

## 1. Amyloid Plaques and Neurofibrillary Tangles in Alzheimer’s Disease

Amyloid plaques and neurofibrillary tangles (NFTs) are pathological hallmarks of Alzheimer’s disease (AD) [1]. Composed primarily of insoluble aggregates of amyloid-β (Aβ) peptides, amyloid plaques are generated through proteolytic cleavage of the amyloid precursor protein (APP). The accumulation of dense-core Aβ structures disrupts synaptic communication, triggers neuroinflammation, and contributes to progressive neuronal loss, key factors underlying the cognitive decline observed in patients with AD [2,3]. The APP is a single-pass transmembrane protein that plays a role in neural development and maturation [4]. Under normal physiological conditions, the APP is primarily processed via the non-amyloidogenic pathway, in which cleavage by α-secretase occurs within the Aβ domain, precluding Aβ formation. However, under pathological conditions such as those observed in AD, the amyloidogenic pathway becomes upregulated. This involves sequential cleavage by the β-site APP-cleaving enzyme 1 (BACE1) and γ-secretase, resulting in the production of Aβ peptides, most notably Aβ40 and the more aggregation-prone Aβ42 species [5]. These peptides tend to misfold and self-assemble into soluble oligomers, which eventually form insoluble fibrils that constitute the core of extracellular amyloid plaques.

As AD progresses, the maturation of Aβ plaques is observed by differential biochemical and morphological characteristics, such as plaque localizations, post-translational modifications, and fibril content [6]. Early-stage plaques are typically diffuse, non-fibrillar, and loosely organized [7]. These diffuse plaques often emerge in the neocortex and may be present in aged individuals without clinical dementia. In contrast, mature plaques, referred to as dense-core or neuritic plaques, exhibit compact fibrillar cores surrounded by dystrophic neurites, reactive astrocytes, and activated microglia. These structures are primarily observed in later stages of AD and are strongly associated with synapse loss and neuroinflammation [8]. These also vary in their size, with diffuse plaques typically ranging from ~10 to >100 µm. Neuritic plaques are ~20–60 µm, while dense-core plaques often measure 50–150 µm in diameter [9].

**Figure 1:**
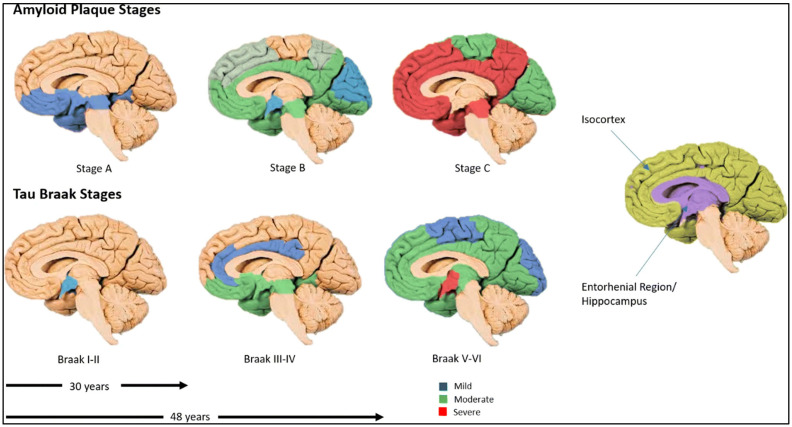
Complementary staging of amyloid-β plaques and tau neurofibrillary tangles (NFTs). Top row (multi-colored brain surfaces) illustrates amyloid plaque accumulation across stages, spreading from cortical association areas into subcortical regions, and eventually cerebellum. Bottom row (multi-colored shading) shows tau tangle progression following Braak stages I–VI, beginning in entorhinal/hippocampal region (in violet shading), and eventually encompassing much of neocortex, in which Alzheimer’s disease symptoms are apparent. Arrows and years indicate the duration it takes to develop Braak stages (from Braak stage I to Braak stage III—30 years; from Braak stage I to Braak stage V—48 years). Reproduced from Swarbrick S et al. [10].

The spatial progression of Aβ deposition in the brain follows a relatively conserved pattern, as shown in Figure 1. In stage A, initial extracellular accumulation of amyloid-β (Aβ) in the basal layers of the frontal, temporal, and occipital isocortex takes place, and this spreads to the isocortical areas in stage B. Finally, dense accumulation of plaques across the isocortex occurs in stage C [10]. These amyloid changes typically precede tau pathology, but do not guarantee later NFT development. However, the emerging evidence supports alternative trajectories in which tau abnormalities may appear first. For example, Arnsten et al. hypothesize that tau abnormalities, particularly in the locus coeruleus, can occur before detectable amyloid accumulation, potentially triggering the cascade that leads to cognitive decline [11]. Another recent data-driven subtyping study identified distinct “amyloid-first” and “tau-first” subtypes using in vivo PET and post-mortem analyses, with the tau-first pattern showing early medial temporal lobe tau accumulation before detectable amyloid, especially among apolipoprotein E (APOE) ε4 non-carriers [12]. These results indicate that tau and amyloid may initiate and spread independently in different subgroups, thus challenging the one-size-fits-all amyloid-first paradigm. Thus, it is imperative to include these perspectives that can significantly affect early AD diagnosis, biomarker interpretation, and therapeutic targeting.

Although Aβ plaques are a defining pathological feature of AD, their precise role in the disease remains unclear. This uncertainty is shown by the limited clinical success of therapeutic interventions that directly target Aβ. Some approaches under investigation include inhibition of β- and γ-secretases to reduce Aβ generation [13], inhibition of Aβ aggregation [14], and enhancement of Aβ clearance through immunotherapeutic or enzymatic strategies [15]. The therapies that target amyloid plaques, including donanemab and lecanemab, have been shown to reduce the amyloid levels in the brain and modestly delay cognitive decline in individuals with early AD. Donanemab, for example, led to significant amyloid clearance in the majority of participants within 18 months and was associated with up to 39% reduction in the rate of clinical decline in those with low-to-moderate tau pathology [16]. Similarly, lecanemab slowed the progression of cognitive symptoms by approximately 27% and reduced the brain amyloid burden over the course of the Clarity AD trial [16]. However, both these treatments carry safety concerns, particularly amyloid-related imaging abnormalities (ARIAs), which can affect up to a third of treated patients. In contrast, aducanumab showed variable results across trials, leading to uncertainty about its clinical value [17]. Overall, while anti-amyloid therapies offer a potential benefit in slowing early disease progression, their use is limited by safety risks, their cost, and the need for early diagnosis. Despite persistent challenges, particularly regarding the optimal timing of intervention and the precise targeting of neurotoxic amyloid-β species, ongoing research into amyloid plaque composition and dynamics continues to enhance our understanding of AD and may inform the design of future therapeutic approaches.

In addition to amyloid plaques, NFTs are another hallmark of AD. While both are defining features of AD, NFTs are characterized by abnormal accumulation of the microtubule-associated protein tau (MAPT), and a group of neurodegenerative diseases characterized by tau aggregation and dysfunction are collectively known as “tauopathies” [18,19]. Tau is a microtubule-associated protein predominantly expressed in neurons, where it plays a critical role in stabilizing the neuronal cytoskeleton. Under physiological conditions, tau binds to microtubules and supports the transport of molecules along axons and dendrites. However, in AD and other tauopathies, tau undergoes abnormal hyperphosphorylation, which reduces its affinity for microtubules and promotes self-aggregation into insoluble filaments. This pathological aggregation leads to the formation of NFTs, which disrupt microtubule function, impair intracellular transport, and ultimately contribute to neuronal dysfunction and cell death. The resulting disruption in neuronal communication is associated with the cognitive deficits seen in dementia, a key clinical manifestation of AD [20]. Hyperphosphorylated tau in NFTs assembles into paired helical filaments (PHFs; ~15–20 nm wide and ~65–80 nm helical crossover) and straight filaments, which accumulate to become micrometer-scale inclusions within neurons [21].

Tau isoforms arise from alternative splicing of the MAPT gene—specifically exons 2, 3, and 10—resulting in six major variants in the adult human brain, distinguished by inclusion of zero, one, or two N-terminal inserts (from exons 2/3) and either three (3R) or four (4R) microtubule-binding repeats (from exon 10). These isoforms are critical for neuronal microtubule stabilization, and imbalances in their 3R:4R ratio are implicated in tauopathies, including AD. Moreover, tau isoform expression is tightly regulated throughout development and across brain regions and cell types, and growing evidence suggests that specific isoforms differentially contribute to neurodegeneration, highlighting the potential for RNA-splicing–based therapies targeting MAPT alternative splicing [22].

Higher levels of NFTs are associated with more severe impairments in memory, attention, and processing speed [23]. NFT pathology in AD advances in a predictable pattern, commonly described by Braak staging, which ranges from stages I to VI (Figure 1). In the earliest stages (I–II), tau protein tangle accumulation begins in the transentorhinal cortex, often before any clinical symptoms emerge. As the disease progresses to stages III–IV, NFTs spread to the entorhinal cortex and the hippocampus—regions crucial for memory—correlating with early signs of cognitive decline. In the later stages (V–VI), tau pathology extends throughout widespread neocortical regions, including the sensory and motor areas, typically accompanying severe cognitive and functional impairment [10]. This gradual progression may unfold over several decades, highlighting a prolonged preclinical phase where molecular changes, such as shifts in microRNA expression, may influence tau phosphorylation, aggregation, and clearance [10]. Such changes may therefore offer valuable insights for early detection. Understanding these stages may aid in identifying molecular and biomarker targets for early diagnosis and therapeutic intervention.

The exact mechanisms by which NFTs propagate through the brain remain unclear. However, the emerging evidence suggests that tau pathology spreads in a prion-like manner. Misfolded tau acts as a “seed” that induces the misfolding and aggregation of native tau in neighboring cells. This cell-to-cell transmission occurs through mechanisms such as exocytosis, endocytosis, and extracellular vesicles, including exosomes [24,25,26]. Additionally, tau has been shown to spread along synaptically connected neurons, facilitating the progression of pathology throughout brain circuits [24].

The tau protein is a central driver of neurodegeneration in AD, with its accumulation in the form of NFTs strongly correlating with disease progression and cognitive decline [27,28]. Therapeutic strategies targeting tau focus on reducing phosphorylation, inhibiting aggregation, enhancing clearance, and stabilizing microtubules. Tau aggregation inhibitors, such as LMTX (leuco-methylthioninium bis(hydromethanesulfonate)), have shown promise by disrupting tau fibril formation, with Phase III trial data suggesting clinical benefits for patients not on concomitant AD medications [29,30]. Antisense oligonucleotides (ASOs) like BIIB080 (IONIS-MAPTRx) reduce tau mRNA expression, effectively lowering the central nervous system soluble tau levels. In a Phase I trial, BIIB080 produced dose-dependent reductions in cerebrospinal fluid (CSF) tau and tau PET imaging signals. Passive immunotherapies, including monoclonal antibodies such as semorinemab and E2814, target extracellular tau species to prevent trans-synaptic spread. E2814 notably reduced the amount of CSF phosphorylated tau by nearly 50% over a two-year period in early AD trials [31]. Novel tau degradation strategies—including proteolysis-targeting chimeras (PROTACs) and nanobody-enabled aggregation disruptors—demonstrate selective clearance of pathological tau, including hyperphosphorylated, misfolded, and aggregation-prone tau species associated with NFTs, in preclinical models [32]. However, major challenges persist: limited blood–brain barrier permeability, modest clinical outcomes despite robust biomarker changes, and an incomplete understanding of the most toxic tau species [33]. Additionally, because NFTs arise in the later disease stages, the therapeutic window may be narrow, potentially limiting the efficacy of tau-directed interventions once neuronal damage becomes irreversible [34].

## 2. Methods Used for Characterization of Amyloid Plaques and Neurofibrillary Tangles

### 2.1. Methods Used for Characterization of Amyloid Plaques

**Figure 2:**
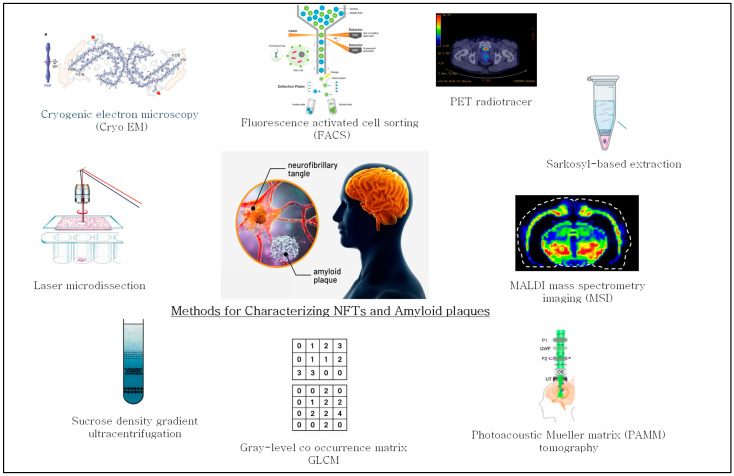
Summary of methods used for characterizing neurofibrillary tangles (NFTs) and amyloid plaques.

The methods used for characterization of amyloid plaques in AD brain tissue have advanced significantly with the integration of histological labeling, laser microdissection (LMD), and high-resolution proteomic tools. These techniques address several challenges, including the heterogeneous nature of plaques, low protein abundance, and variable tissue preservation. Despite this progress, limitations remain, prompting the development of more refined strategies for isolating and characterizing amyloid plaques. A summary of selected methodologies for amyloid plaques enrichment and analysis is presented in Table 1 and Figure 2.

A widely adopted strategy used for studying amyloid pathology involves the use of formalin-fixed paraffin-embedded (FFPE) brain sections, which preserve both tissue morphology and structural integrity. Immunohistochemistry (IHC) is commonly utilized either before microdissection or as a post-analytical validation step to accurately localize amyloid-β (Aβ) deposits [35]. The classical staining methods include the use of thioflavin-S, a dye that selectively binds β-sheet-rich fibrillar amyloid structures [36]; Congo red is another major staining method that is used to detect the amyloid structure of protein aggregates [37], and anti-Aβ antibodies [38]. These markers allow for precise identification of amyloid plaques and help guide subsequent microdissection procedures.

A particularly refined approach was described by Xiong et al. (2019), who used the fluorescent dye Amylo-Glo^®^ RTD™ with LMD to capture amyloid plaque-containing regions and adjacent cortical regions from brain tissue (Table 1) [39]. LMD is a precision technique used to capture targeted cells or tissue regions—from entire areas down to single cells—using a microscope-guided laser system. The laser cuts around the region and catapults it to a collection vessel or melts an adhesive film to capture the laser microdissected tissue, maintaining both morphological and molecular integrity for downstream analyses like DNA/RNA sequencing and proteomics [25]. Laser capture microdissection employs a laser to melt the target tissue onto a plastic substrate, enabling its separation from the tissue slide [40]. Recently, automated LMD enabled precise capture of single cells based on AI-guided morphological segmentation, allowing for high-throughput spatially resolved proteomic profiling [41]. In addition, a recent advanced approach integrated highly multiplexed imaging, single-cell LMD, and sensitive mass spectrometry to spatially profile the proteomes of distinct cell populations in human cancers with high sensitivity [42]. These approaches enhanced accuracy and reproducibility, but remain limited in broader adoption due to their high costs, technical complexity, and the need for specialized expertise. As a result, its use is largely restricted to advanced research facilities with integrated imaging and proteomics infrastructure.

Using LMD, Xiong et al. microdissected around 3000 plaque-containing areas per brain, followed by protein extraction, trypsin digestion, tandem mass tag (TMT) labeling, and liquid chromatography–tandem mass spectrometry (LC-MS/MS) analysis for comprehensive proteomic profiling [39]. Circular regions with 80–100 µm diameter were centered on amyloid plaques and were collected, and for each a corresponding non-plaque region of identical size was sampled from the surrounding cortex within 500 µm of the plaque edge and was used as a control. While the method provides spatial precision with precise capturing of plaque-containing regions, it has several limitations, including a small sample size and a cross-sectional design. Due to the cross-sectional design, whereby tissue was analyzed from a single time point per subject, it did not show whether certain proteins accumulate earlier or later in plaque development, or how the proteomic profile evolves as the disease progresses. Additionally, the study lacks functional validation, leaving the roles of identified proteins in disease mechanisms unresolved [39]. Chemical noise from polyethylene naphthalate (PEN) oligomers in mass spectra acquired from LMD-collected samples remains is another concern for mass spectrometry analysis [43,44].

**Table 1:** Comparison of methodologies for characterizing amyloid plaques in Alzheimer’s disease (AD). Methods described include laser microdissection (LMD)-based proteomics [39], sucrose density gradient ultracentrifugation [45], texture-based imaging analysis using gray-level co-occurrence matrices (GLCMs) [46], MALDI mass spectrometry imaging (MSI) [47], and photoacoustic Mueller matrix (PAMM) [48]. Advantages and limitations of each method are briefly summarized.

Method	Material	Advantages	Limitations	Reference
Laser microdissection of amyloid plaque-containing regions from AD brain sections, controls, and APP/PS1 transgenic mice.	Amyloid Plaque regions and adjacent non-amyloid plaque regions.	Cross-species comparison. Downstream Analysis. Revelation of protein increase in both AD and aging.	Time-consuming LMD. Small Sample Size (*n* = 3 per sample group) and a cross-sectional design limit. Potential contamination. Underrepresentation of low-abundance or hydrophobic proteins. Translation of mouse study to human pathology.	[39]
Sucrose density-gradient ultracentrifugation.	Postmortem brain tissue.	High purity. Reproducible. Scalable. Quantitatively robust.	Laborious. Contamination Risk.	[45]
Gray-Level Co-occurrence. Matrix (GLCM) texture analysis. Brain tissue samples from Alzheimer’s disease (AD) and non-AD individuals were immunostained for amyloid-β.	Graphic processing of Aβ-stained plaques using GIMP software v.2.10.	High Throughput and Non-Destructive. Objective Quantification. Reproducible.	Indirect Measure (texture analysis, not biochemical analysis). Dependent on Staining/Imaging Quality. Requires Computational Expertise.	[46]
MALDI mass spectrometry imaging (MSI). 2D and 3D-MSI analysis.	2D and 3D imaging of amyloid plaques followed by computational evaluation and quantitation.	Automated, pixel-level plaque detection. Heterogeneity profiling across models. Single plaque quantitative metrics. Accurate 3-D reconstruction with elastic registration.	Requires serial sections. Applicability to human brain tissue or FFPE samples remains unproven. Low spatial resolution. Relative quantification due to matrix effects and ion suppression. Computationally demanding. Lack of standardized workflows challenges reproducibility and clinical translation.	[47]
Photoacoustic Mueller matrix (PAMM) tomography Label-free imaging technique. Uses polarization-sensitive optical absorption to visualize amyloid-β plaques in 3D.	Brains of APP/PS1 Alzheimer’s mouse models.	Completely label-free. Quantitative 3D imaging.Molecular conformation.	Validation in human and clinical applicability remains uncertain.Complex method may challenge reproducibility.	[48]

An alternative biochemical approach to enriching amyloid plaques from bulk tissue relies on the differential solubility and sucrose density of amyloid aggregates. A recent article described a robust, label-free workflow for isolating amyloid-β plaque cores from postmortem brain tissue [45] (Table 1). The method begins with homogenization and sequential filtration to remove cells and debris, followed by sucrose density-gradient ultracentrifugation to enrich plaque fractions. After collecting the dense layer (~1.6 M sucrose), researchers perform detergent washing (e.g., SDS), re-centrifugation, and optional lyophilization before biochemical or microscopy assessments. The method enables high-purity plaque enrichment, while preserving β-sheet structures, ideal for downstream proteomics or imaging. The method is scalable, reproducible, and quantitatively robust, but is labor-intensive, requiring multiple rounds of centrifugation, which can introduce variability and increase the contamination risk.

Beyond biochemical and histological techniques, computational image analysis has emerged. Zaletel et al. (2021) use gray-level co-occurrence matrix (GLCM) texture analysis to distinguish the plaque morphologies associated with AD from those found in individuals without AD (Table 1) [46]. Although this approach does not help isolate plaques, it employs a novel method to differentiate plaque types based on morphologies and texture heterogeneity. GLCM analysis identifies specific pixel intensities and analyzes the differences between neighboring pixels [46]. This approach builds on the observation that dense-core plaques, more commonly associated with cognitive impairment, exhibit greater structural complexity compared to diffuse plaques, which may occur in cognitively normal elderly individuals [48]. Using GLCM texture analysis, the detection of the plaque type could serve as a valuable method to investigate AD association. In their study, 1039 plaques from 69 patients with and without AD were digitally analyzed, revealing significantly higher heterogeneity in the AD-associated plaques [46]. The increased texture variability may reflect underlying biochemical differences, such as differential protein aggregation and spatial orientation [9]. GLCM, along with a machine learning framework extracted from structural MRI scans, is useful to classify AD, mild cognitive impairment (MCI), and healthy controls. The approach has been shown to achieve high classification accuracy (up to 98.5% for AD vs. MCI), demonstrating GLCM’s effectiveness in capturing the subtle brain tissue texture changes associated with neurodegeneration [49].

MALDI mass spectrometry imaging (MSI) enables label-free, spatially resolved analysis of a wide range of analytes in tissue sections. It preserves the spatial and morphological context, while acquiring detailed molecular information from biological samples, albeit within a limited dynamic range. The laser damages the top layer of the tissue, and after the analysis, the tissue is not usable for some downstream applications such as RNA analysis, but it is often still usable for histology. MSI offers powerful, label-free spatial mapping of biomolecules in tissues, but several limitations hinder its broader adoption. Its spatial resolution (10–100 µm), while improving, often falls short of single-cell precision, and the technique typically requires time-intensive acquisition and complex sample preparation. MSI is typically optimized for specific molecular classes (e.g., lipids, metabolites, and peptides) as it is difficult to image all biomolecule types simultaneously with the same method. [41]. The 3D plaque MSI tool [47], Table 1, enhances compositional analysis by revealing molecular subtypes of Aβ plaques, often missed in bulk studies. Aligning serial 2D MALDI-MSI sections creates high-resolution 3D maps, revealing small plaques and detailed peptide distributions. The automated “plaque picker” enables fast, precise, unbiased detection and quantification, providing reproducible metrics like plaque volume and peptide patterns for more accurate technical and biological comparisons.

A recent study introduced a label-free photoacoustic Mueller matrix (PAMM) tomography imaging technique that exploits polarization-sensitive optical absorption to visualize amyloid-β plaques in 3D, deep within the brains of APP/PS1 Alzheimer’s mouse models [48] (Table 1). This method enables in vivo, depth-resolved detection of plaques without dyes or labels, capturing new molecular conformation parameters across the different disease stages. The advantages include being completely label-free, which avoids the need for fluorescent markers or stains. It offers in-depth information and spatial localization of plaques via quantitative 3D imaging and provides biochemical insights beyond the mere structure. However, the complexity in photoacoustic polarization measurements may challenge the reproducibility of the method. In addition, validation in human tissue and comparison with conventional imaging (e.g., PET and MRI) remains uncertain.

While techniques such as LMD-guided mass spectrometry and density-based separation offer detailed biochemical insights, texture analysis presents a novel method to correlate plaque morphology with disease pathology. Continued development of hybrid methods that combine spatial resolution with proteomic depth will likely improve our understanding of plaque heterogeneity and progression of AD.

### 2.2. Methods Used for Characterization of Neurofibrillary Tangles (NFTs)

Over the years, various techniques have been developed to extract and analyze NFTs, key features of AD, and tauopathies. Formalin-fixed paraffin-embedded tissue is commonly used for the preservation of structural detail [50]. NFTs are typically visualized using immunohistochemistry or immunofluorescence [38] with anti-phospho-tau antibodies, alongside classical silver stains like Gallyas [51], Bielschowsky [9].

Campbell-Switzer[9].and Bodian [52]. For studying NFTs, approaches such as LMD, Sarkosyl-based extraction [48], immunopurification, density gradient centrifugation, and FACS [48] are widely used to enrich, selectively capture, and analyze NFT-containing material. A summary of methods used for the characterization of NFTs is shown in Table 2 and Figure 2.

Advancements in analytical methods have significantly enhanced the ability to characterize NFTs at the molecular level. Proteomic and genomic approaches [57,58], as well as bioimaging and stereological analysis, are now regularly applied to study the composition, distribution, and functional impact of NFTs. Despite these advances, each method carries inherent advantages and limitations. For example, while immunopurification offers specificity, it may result in loss of associated protein complexes. Similarly, techniques like LMD provide spatial precision, but can be labor-intensive and yield limited material. Overall, the continued refinement of NFT enrichment, characterization, and analysis methods is essential for understanding AD pathology and identifying potential therapeutic targets in neurodegenerative disease.

Fluorescence-activated cell sorting (FACS) is a non-denaturing, fluorescence-based technique used to sort and enrich cell populations containing tau aggregates by labeling them with specific antibodies targeting tau aggregates. First applied to NFTs by Hussey et al. (1986), FACS enables high-purity separation of tangle-containing neurons from brain tissue. Recent studies have used FACS combined with antibodies recognizing different tau conformations to isolate NFT-positive neurons from AD brains for detailed transcriptomic and proteomic analyses [53] (Table 2). This method preserves antigen integrity and allowssingle-cell resolution. However, FACS is time-intensive and sensitive to tissue quality, with potential contamination from co-localized amyloid plaques. Despite these challenges, FACS remains a powerful method for selectively enriching cell populations containing pathological tau, complementing other biochemical methods to advance understanding of tau-related neurodegeneration.

Sarkosyl-based extraction is a widely used technique for extracting NFTs by selectively enriching detergent-insoluble protein aggregates, particularly pathological tau associated with AD [54] (Table 2). As described by Diner et al., frozen brain tissue is homogenized in a low-salt-content buffer containing protease and phosphatase inhibitors to preserve protein integrity. Sarkosyl and NaCl are then added to solubilize the native, soluble proteins. The homogenate undergoes sonication and ultracentrifugation, which separates soluble proteins in the supernatant from detergent-insoluble aggregates in the pellet. The pellet, enriched in aggregated and phosphorylated tau species, can be washed and solubilized in a urea-containing buffer for downstream biochemical and proteomic analyses, including mass spectrometry. This method offers advantages, such as speed, simplicity, preservation of aggregate ultrastructure, and compatibility with comparative studies between AD and control samples. However, sarkosyl extraction may co-extract other insoluble components, resulting in reduced purity, and the heterogeneity of aggregates can complicate data interpretation. Additionally, it does not readily differentiate between tau isoforms or aggregation states without further purification. Overall, sarkosyl-based extraction remains a powerful and efficient approach for enriching NFTs, facilitating detailed molecular and structural characterization of tau aggregates in neurodegenerative research.

A recent advancement in non-invasive NFT analysis PET imaging employed the tau-specific radiotracer ^18^F-MK-6240, which has high selectivity and sub-nanomolar affinity for aggregated tau proteins [55] (Table 2). Lohith et al. performed dynamic PET imaging by administering ^18^F-MK-6240 intravenously to ten participants (four healthy controls, four people with AD, and two with amnestic MCI). The ^18^F-MK-6240 PET revealed increased tracer uptake in tau-rich regions of the patients with AD and MCI, consistent with known tau accumulation patterns. The brain areas exhibiting elevated uptake of ^18^F-MK-6240—a PET tracer with high affinity for NFTs composed of aggregated tau—were labeled as tau-rich regions. Using the cerebellar cortex as a reference and advanced kinetic modeling, the study quantified tracer binding, while arterial sampling distinguished the parent compound from the metabolites. The advantages include high sensitivity, specificity, and suitability for longitudinal monitoring. The limitations include small sample size, occasional off-target or atypical binding, and challenges in detecting early-stage tau. Overall, ^18^F-MK-6240 PET provides a powerful tool for in vivo detection, mapping, and quantification of abnormal tau accumulation—particularly NFTs—which are characteristic of AD and related tauopathies, with potential applications in early diagnosis, disease staging, and therapy assessment.

A recent method used a cultured cell-based model and sarkosyl enrichment of insoluble tau with the cryogenic electron microscopy (cryo-EM) method [59] (Table 2). Cryo-EM enables high-resolution 3D visualization of biomolecules and cellular structures by flash-freezing samples in vitreous ice and imaging them with an electron beam at cryogenic temperatures [56]. Unlike X-ray crystallography, it does not require crystallization and preserves specimens in their near-native states. In this study, the researchers seeded undifferentiated neuroblastoma cells expressing human tau with brain-derived extracts from patients with AD or corticobasal degeneration (CBD). They then extracted the resulting sarkosyl-insoluble tau filaments and determined their structures via cryo-EM. This method provided structural insights into templated tau propagation by revealing how filament structure is influenced by disease-specific seed conformations. The cell-based model enables manipulation of tau isoform expression and seeding conditions and provides high-resolution structural determination suitable for atomic modeling. But there are certain limitations associated with this method. The filament structures differ from brain-derived seeds, indicating incomplete templating. Cultured cell environment may lack critical cofactors or post-translational modifications present in vivo, and undifferentiated cells may not represent neuronal complexity, potentially affecting pathophysiological relevance. In addition, cryo-EM is a high-cost and technically complex technique that involves intricate workflows and data processing.

As summarized in Table 2 and Figure 2, the methods discussed share a common methodological framework: they target NFTs based on their unique biochemical and structural properties to enrich, detect, or quantify them in complex biological samples. These techniques—ranging from IHC and LMD to sarkosyl-based extraction, FACS, and PET imaging—offer complementary strengths in visualizing, enriching, and characterizing NFTs. While each method contributes critical insights—such as spatial resolution, structural detail, and in vivo mapping—they also exhibit specific limitations, including issues with scalability, biochemical depth, throughput, and resolution. Therefore, these methodologies are often best used in combination. To overcome the individual limitations and achieve a more comprehensive characterization of NFTs and tau, integration with advanced analytical techniques, such as mass spectrometry, proteomics, and single-cell omics, would enhance molecular profiling, quantification, and scalability, providing deeper insight into the composition, post-translational modifications (PTMs), and progression of NFTs across different stages of neurodegeneration.

## 3. Mass Spectrometry-Based Proteomic Studies of Amyloid Plaques and Neurofibrillary Tangles in Alzheimer’s Disease

### 3.1. Mass Spectrometry-Based Proteomics Studies of Amyloid Plaques

Mass spectrometry (MS) has become a vital technique for analyzing the complex protein composition of amyloid plaques, which are key pathological features of Alzheimer’s disease (AD). MS-based proteomics allows for sensitive and comprehensive identification and quantification of proteins, shedding light on the molecular components and post-translational modifications present within amyloid plaque-containing regions. This method has deepened our understanding of the variety of proteins present within plaque-containing regions, including amyloid-beta peptides and other associated molecules that may influence plaque toxicity. Advances in quantitative MS approaches, such as TMT and label-free quantification, have improved comparisons of protein levels across different brain regions and disease stages. These studies play an important role in uncovering the biochemical mechanisms of AD and may guide the development of new therapeutic strategies. Table 3 outlines the recent mass spectrometry-based proteomics studies of amyloid plaques.

Xiong et al. conducted a comprehensive proteomic analysis of amyloid plaque-containing regions from human AD brains, aged non-AD controls, and transgenic mouse models using LMD, TMT labeling, and high-resolution LC-MS/MS (Table 3) [39]. They profiled over 3000 plaque regions per brain and identified more than 4000 proteins, significantly increasing the proteome coverage compared to that of previous studies [63,64]. The key proteins enriched in the human AD plaque-containing regions included APCS, LRP1, Tomoregulin-1, CCT4, and Mcu, involved in different biological pathways like endocytosis, chaperone activity, and mitochondrial regulation. The study utilized Amylo-Glo^®^ RTD™ staining and high-performance liquid chromatography (HPLC) peptide separation to distinguish the proteins linked to normal aging versus AD pathology, finding no direct correlation between chronological age and plaque proteome, but noting an age-associated increase in the number of non-AD plaque proteins. Despite the challenges in peptide quantification due to protein modifications, consistent enrichment patterns were identified. Overall, this study highlights distinct amyloid plaque-enriched proteome profiles of human AD, aged non-AD controls, and APP/PS1mouse models, which may relate to different pathological processes.

Another study utilized LMD to collect amyloid plaque-containing regions, followed by ultra-sensitive data-independent acquisition (DIA) mass spectrometry-based proteomics [62] (Table 3). Frozen brain sections (10–12 µm thick) were fixed with 70% alcohol and stained with X-34 dye, and then one plaque and one non-plaque containing area (approximately 50 µm diameter) from the same sections were captured using LMD. The proteins were extracted from the captured tissue regions by using a specialized 0.2% DCA buffer, minimizing sample loss as no desalting step was required. The work identified around 20,000 peptides corresponding to approximately 5000 various proteins, including hallmark disease-related proteins, such as Aβ, ApoE, Mdk, and Ntn1. The study provided a unique method combining LMD coupled with DIA proteomics, providing insight into plaque-associated molecular composition in AD [62].

Drummond et al. applied proteomic analyses to formalin-fixed, paraffin-embedded brain tissues from patients with AD and Down syndrome, both exhibiting amyloid-beta (Aβ) accumulation [60] (Table 3). IHC was used to distinguish the plaques from the non-plaque regions, followed by LMD to collect a 2 mm^2^ area of fluorescently labelled plaques and neighboring non-plaque containing regions. The tissues were processed to extract peptides and perform LC-MS/MS analysis. The study identified 48 proteins enriched in plaque-containing regions, including 15 novel candidates. A 1.5-fold change threshold was applied to define enrichment. Among the most enriched were SMOC1, COL25A1, APCS, and OLFML3. SMOC1, a matricellular protein known to interact with Aβ and phosphorylated tau, showed significant enrichment and colocalization with plaques containing post-translationally modified Aβ species [65,66]. Serum amyloid-P (APCS) is a universal amyloid-associated protein that binds amyloid fibrils in a calcium-dependent manner, stabilizing them against proteolysis and potentially prolonging plaque persistence. Proteomic studies consistently detect APCS as highly enriched in AD plaques, supporting its role as a structural and possibly pro-inflammatory component [39,60]. COL25A1 is the most enriched protein in early-onset AD, as shown in this study [60], and late-stage AD, as shown in Xiong et al.’s study [39], thus pointing to its potential role in late AD progression [44]. Immunofluorescence confirmed SMOC1 colocalization with only a subset of plaques containing modified Aβ, suggesting its involvement in early AD pathology, consistent with the early occurrence of Aβ phosphorylation. This study highlights novel plaque-associated proteins and suggests stage-specific roles in AD progression.

Levites et al. conducted an integrative proteomic study to define the Aβ “responsome”—a network of proteins that co-accumulate with Aβ—in both human AD brains and CRND8 transgenic mouse models [61] (Table 3). Using bottom-up proteomics, the study revealed age-dependent and Aβ-correlated protein expression changes. Notably, two heparan sulfate-binding proteins, midkine (Mdk) and pleiotrophin (PTN), were identified as co-localizing with Aβ plaques in both species. Functional assays showed that in vivo overexpression of Mdk and PTN increased the fibrillar Aβ levels, while in vitro Thioflavin T (ThT) assays confirmed their ability to accelerate Aβ42 aggregation. These findings suggest that Mdk and PTN may actively promote Aβ pathology, providing mechanistic insight into their potential roles in disease progression and highlighting targets for therapeutic intervention.

A proteomics method developed by Drummond et al. (2018) used FFPE human brain tissue, where amyloid plaque-containing regions were identified by immunofluorescence, captured via LMD, and analyzed using label-free quantitative LC-MS/MS [38] (Table 3). This approach enabled the identification of approximately 900 proteins from a 2 mm^2^ area from 8 µm thick sections of plaque-enriched regions, including known components, such as Aβ, ApoE, clusterin, and ubiquitin. Unlike antibody-based methods, this unbiased strategy provided a more comprehensive and spatially resolved proteomic profile, facilitating the discovery of novel plaque-associated proteins. In summary, these studies have identified numerous novel proteins, such as SMOC1, Mdk, and PTN, that are potentially involved in plaque development and progression. By dissecting the molecular complexity of plaques across different stages of AD, MS proteomics continues to refine biomarker discovery and guide the development of targeted therapeutics.

### 3.2. Mass Spectrometry-Based Proteomics Studies of Neurofibrillary Tangles (NFTs)

NFTs, primarily composed of hyperphosphorylated tau protein, have traditionally been studied using biochemical and histological techniques, which provide structural and localization data, but limited insight into molecular composition. To gain a deeper understanding of the proteins and modifications involved in NFT pathology, MS-based proteomics enables comprehensive identification and characterization of proteins, including PTMs such as phosphorylation, acetylation, and truncation, key processes in tau aggregation and NFT formation. As reviewed by Cui et al. (2022) [67], proteomic workflows allow for the analysis of intact proteins and isoforms, mapping protein networks, and identifying biomarkers with potential diagnostic or therapeutic value. The MS-based approach outlined in their workflow moves from enriching tau species and associated proteins to conducting network-level analyses, ultimately aiding in biomarker discovery. This strategy not only expands our molecular understanding of NFTs, but also supports efforts in therapeutic development and disease monitoring.

Pichet Binette et al. (2024) utilized a high-throughput proteomic approach combining the Olink Explore 3072 panel with tau PET imaging to identify (CSF proteins associated with in vivo tau tangle accumulation [68] (Table 4). Analyzing the CSF from 877 individuals in the BioFINDER-2 cohort, they employed the Olink proximity extension assay—a multiplexed immunoassay that uses next-generation sequencing (NGS) tags—to quantify thousands of proteins. The participants were stratified by the Aβ42/40 ratio and tau PET into groups with no pathology, isolated amyloid pathology, and combined amyloid and tau pathologies. Although NFTs were not directly accessed, CSF proteomics analysis identified 127 proteins that varied significantly across these groups. Several proteins, including MAPT (tau), FABP3, ENO1/2, MIF, NRGN, and GLOD4, were strongly correlated with the tau PET signal and implicated in neuronal injury, metabolic stress, synaptic transmission, and mitochondrial dysfunction. This approach demonstrates the potential of CSF proteomic profiling to investigate molecular changes across disease stages, which may support future efforts in biomarker discovery.

An effective method for analyzing NFTs involves combining sarkosyl-insoluble fraction extraction with LC-MS/MS to identify tau PTMs. Detergent-insoluble fractions enriched in the NFTs were obtained from homogenized brain tissue of patients with AD. These fractions were subjected to trypsin digestion, and the resulting peptides were analyzed by LC-MS/MS. Using a comprehensive proteomic workflow and bioinformatics tools, including Proteome Discoverer, Mascot, Scaffold, Swiss-Prot, GenBank, and jPOST [69] (Table 4), the researchers mapped 170 distinct PTMs across tau, including phosphorylation, ubiquitination, acetylation, and methylation. The PTMs were concentrated in functionally critical regions, such as the proline-rich and microtubule-binding domains, especially between amino acids 181 and 238. This molecular fingerprinting highlighted PTMs associated with tau misfolding and aggregation in AD. The method offers high sensitivity and can detect multiple PTM types with site-specific resolution, providing valuable mechanistic insights. However, the limitations include the potential for sample processing artifacts, dependence on high-quality tissue, and challenges in interpreting complex PTM patterns.

A label-free proteomic method developed by Drummond et al. (2018) (Table 4) [38] (Table 4) combined LMD with LC-MS/MS to directly analyze amyloid plaques (as mentioned in the amyloid plaques MS section) and NFTs from FFPE post-mortem human brain tissue. The study identified approximately 500 proteins from a 1.5 mm^2^ NFT-enriched area from 8 µm thick tissue sections, including phosphorylated tau, heat shock proteins, kinases, and cytoskeletal elements. This method enabled unbiased detection of both known and novel, low-abundance proteins directly from pathological lesions. While powerful, the approach is limited by the labor-intensive nature of LMD and challenges associated with protein extraction from FFPE tissue due to crosslinking and fixation induced modifications.

A recent proteomic study employed differential detergent-insoluble fractionation combined with a novel complement-adjusted TMTc mass spectrometry approach to the examine postmortem frontal cortexes from five patients with AD and five matched controls [70] (Table 4). Out of more than 8900 proteins detected, 190 were significantly expressed in AD, with 84 definitively enriched in the insoluble fraction, highlighting the aggregated species, such as tau, amyloid-β, TDP-43, U1-70K, MDK, PTN, NTN1/3, and SMOC1. TDP-43, in particular, co-aggregated with tau and Aβ in detergent-resistant fractions and was implicated in RNA-splicing dysfunction through enrichment of U1 snRNP proteins, supporting previous evidence linking its aggregation to mild cognitive impairment, AD, and tau pathology [74]. Functional pathway analysis revealed disruptions not only in amyloid processing and protein degradation, but also in endocytosis/exocytosis, RNA metabolism, and synaptic signaling. The comprehensive TMTc workflow, validated via immunoblotting, provides high-resolution, quantitative profiling of sarkosyl-insoluble, tau-enriched protein fractions from AD brain tissue, revealing both known and novel proteins associated with disease-relevant aggregates. While the study’s methodology offers rigorous aggregate detection and broad proteomic coverage, it is inherently limited by reliance on postmortem tissue, potential contaminants in detergent-insoluble fractions, and the need for large-scale validation across diverse brain regions and disease stages.

Another study by Drummond et al. employed a dual proteomic strategy to analyze the proteins within NFTs containing regions and those interacting with phosphorylated tau (p-tau) (Table 4) [71]. The brain regions enriched in NFTs were laser microdissected from post-mortem brain tissue after staining with the AT8 antibody. A 1.5 mm^2^ area consisting of ~4000 NFTs per sample was collected. LC-MS/MS analysis of AT8-positive, NFT-enriched neurons identified around 542 proteins, including tau, ubiquitin, neurofilaments, and ApoE. In parallel, affinity purification mass spectrometry (AP-MS) with the PHF1 antibody was used to immunoprecipitate p-tau and its binding partners from brain homogenates. This revealed 75 p-tau interactor candidates, including 29 previously linked to p-t and 34 known tau-associated proteins like VAMP2 and PURA. The approach uncovered 12 novel proteins, such as RNA-binding protein HNRNPA1, that had not previously been known to be physiologically or pathologically associated with tau. This combined method offers high spatial specificity and direct identification of tau interactor candidates, but is limited by poor tissue quality and the potential to miss weak or transient interactions.

A recent study analyzed tau from both soluble (TBS) and sarkosyl-insoluble (SI) fractions of human frontal cortex across AD, progressive supranuclear palsy (PSP), Pick’s disease (PiD), corticobasal degeneration (CBD), and controls using immunoprecipitation and high-resolution mass spectrometry with isotope-labeled standards [72] (Table 4). They found that 0N and 1N tau isoforms were most abundant overall. In the SI fraction, the AD samples showed a higher level of 0N tau isoform. The PiD samples were mainly composed of 3R tau isoform, while PSP and CBD predominantly exhibited 4R tau, with AD and controls showing mixed 3R/4R patterns. The SI fractions, particularly in AD, were enriched in peptides from the microtubule-binding region, which are known to be more likely to form aggregates. Additionally, the SI fractions exhibited higher phosphorylated peptides than soluble fractions, with AD showing especially elevated levels, even in the soluble pool. This work provides isoform- and PTM-specific profiles across tauopathies, highlighting disease-specific signatures, notably multi-phosphorylation in AD.

A quantitative proteomics study analyzed the detergent-soluble and insoluble fractions from AD brain tissue to characterize tau and Aβ species by biochemical assays, electron microscopy, and targeted mass spectrometry analysis [73] (Table 4). The sarkosyl-insoluble fraction was enriched in amyloid-β42 and truncated tau peptides, particularly from the microtubule-binding region, which were more abundant than full-length or N-terminal tau. Disease-relevant phosphorylation sites like threonine 181 and 217 were also elevated in this aggregated fraction. Structural imaging revealed both early globular aggregates and mature tau filaments coexisting, suggesting a progression between forms. These results underscore the biochemical and structural heterogeneity of AD aggregates and indicate that specific tau fragments and modifications may drive disease progression. However, the use of postmortem homogenates limits spatial resolution and may capture non-pathological proteins within aggregates.

MS-based studies have provided deep insights into the molecular complexity of tau and NFTs in AD. They have revealed a rich landscape of tau isoforms, post-translational alterations, aggregate-associated proteins, and emerging fluid biomarkers, yet integrating spatial, longitudinal, and functional dimensions in larger studies remains a key challenge. Together, MS-based proteomics has established NFTs and tau as both structurally and functionally diverse, offering critical leads for diagnostics and therapeutic targeting. It is vital to consider that some biomarkers, particularly those measured in CSF or plasma, may have a peripheral contribution. For example, Pichet et al. (2024) [68] analyzed CSF proteomics alongside PET imaging; although these proteins primarily reflect CNS pathology, peripheral contributions cannot be entirely excluded. In contrast, studies examining postmortem brain tissue or detergent-insoluble fractions [60,70,72,73] are largely unaffected by peripheral sources, as the proteins analyzed are tissue-localized. Considering potential peripheral contributions in fluid-based measurements is therefore crucial for accurately interpreting disease-specific molecular changes.

## 4. Summary and Discussion

Label-free, TMT, and modern DIA proteomic profiles of Aβ plaques and NFTs at a high spatial resolution uncovered both well-characterized and novel associated proteins. Approaches such as single-plaque DIA-MS have further enhanced proteomic depth, though they remain technically demanding. In contrast, bulk extraction or biochemical extraction (such as sarkosyl)-based proteomics provides broad molecular coverage, but lacks the cellular and anatomical precision preserved in LMD-based workflows. Integrating both spatially resolved and bulk strategies can offer complementary insights into AD pathology, combining the chemical depth of bulk proteomics with the spatial granularity of lesion-level analysis to better understand amyloid and NFT biology. In addition, some of the other emerging approaches, including blood-based biomarkers such as phosphorylated Tau181 [75] and brain-derived extracellular vesicles [76], offer non-invasive, longitudinal measures of disease progression. Integrating these methods with the existing proteomic analyses could enhance patient characterization by enabling early detection, monitoring temporal dynamics, and stratifying individuals based on molecular profiles, ultimately improving the interpretability and clinical relevance of proteomic findings.

However, the mere presence of proteins and their PTMs within a plaque or NFTs does not in itself establish a causal or mechanistic role in AD pathology. Many detected species may be co-localized due to nonspecific trapping, age-related changes, or the regional proteome context rather than direct pathogenic relevance. Thus, extensive follow-up studies with larger cohorts and orthogonal validations in independent cohorts, biochemical functional assays, and longitudinal clinical correlation are imperative to determine whether any of these proteins or PTMs are consistently associated with AD progression or hold promise as reliable biomarkers for diagnosis, prognosis, or therapeutic monitoring.

ApoE is a key genetic and biochemical factor in AD, with its isoforms (ε2, ε3, and ε4) differentially modulating amyloid-β aggregation, tau pathology, and neuroinflammatory responses. The ApoE isoforms likely influence proteomic profiles of both amyloid plaques and NFTs. Studies using LMD and MS [38,39,60,61,68] consistently identify ApoE in plaques, with ApoE4 associated with increased Aβ deposition and altered plaque composition. Similarly, tau proteomics studies [69,70,71,72,73] suggest that ApoE isoforms can modulate tau phosphorylation, isoform distribution, and tau-associated protein networks. Thus, considering the ApoE genotype in proteomic analyses is critical for interpreting isoform-dependent differences in protein composition, PTMs, and aggregation patterns in AD.

In addition, patients with double PSEN1 mutations, such as K311R and E318G, exhibit distinct proteomic profiles compared to typical patients with AD. These mutations can alter γ-secretase activity, affecting amyloid precursor protein processing and the Aβ peptide ratios detectable in CSF or brain tissue. Proteomic analyses reveal dysregulation of proteins involved in synaptic function, neuroinflammation, and extracellular matrix remodeling, reflecting the combined effects of the mutations. Profiling these changes can provide biomarkers to identify double-mutation carriers and offer insights into their disease mechanisms, enabling early detection and targeted interventions, even without genetic testing. A recent study identified 66 significantly dysregulated proteins in the CSF of familial AD mutation carriers, highlighting the value of proteomic analyses in understanding disease pathophysiology [77]. Integrating proteomic data with genetic screening can further enhance identification, facilitate early diagnosis, and support personalized therapeutic strategies for patients with complex genetic profiles.

A careful consideration of pre-analytical variables, such as the postmortem interval (PMI), formalin fixation, and the sample processing conditions, is necessary for accurate proteomics data interpretation. A long PMI can lead to protein degradation, including proteolysis, oxidation, and deamidation, which can interfere with true disease-associated alterations in disorders like AD. Studies have shown that a longer PMI alters protein composition significantly, with substantial degradation becoming apparent after 24–48 h [78]. A recent study utilizing a gel-based approach to profile changes in the mouse brain cortex at different PMIs identified potential new biomarkers useful for PMI assessment. It provided additional parameters for quality controls, emphasizing the need for PMI-matched controls to distinguish genuine pathology-related changes from artifacts [79]. Similarly, formalin fixation induces methylene crosslinks between proteins, impeding protein extraction and enzymatic digestion. Thus, it is necessary to employ methods to reverse formaldehyde-induced crosslinking, such as heat-induced antigen retrieval, to improve the accessibility of epitopes for downstream analysis. Recently, an optimized protocol utilizing a buffer containing Tris, SDS, SDC, and Triton X-100 has been shown to enhance protein extraction efficiency and proteome coverage [80]. Thus, to reliably associate protein presence and abundance with disease states, it is crucial to implement standard protocols for sample handling and processing, as well as appropriate age-, sex-, and PMI-matched non-diseased controls processed under similar conditions to minimize variability and enhance reproducibility of proteomic studies.

Noticeably, not all the methods that characterize plaques and NFTs measure the same biological features, and the choice of technique can significantly impact their proteomic profiles. Proteomic analyses by LMD can capture both fibrillar Aβ and associated extracellular matrix, immune, and synaptic proteins. In contrast, biochemical extractions often deplete loosely associated or peripheral components, such as extracellular matrix proteins, resulting in markedly different proteomic profiles [38,39,69]. Similarly, FFPE-based workflows may under-represent specific proteins due to fixation and crosslink-reversal chemistry, producing differences from fresh-frozen or extracted plaques or NFTs. A recent study identified a higher number of AD-related proteins in fresh frozen tissues, which were not identified in prior FFPE preparations [81]. In addition, in vivo imaging such as PET and ex vivo approaches such as IHC can target different Aβ and NFT conformers or associated proteins, often reflecting biologically distinct pools of amyloid and tau pathologies [46,47,55].

Proteomics of plaques and NFTs focuses specifically on proteins, and thus misses other essential biomolecules, such as glycosaminoglycans (GAGs), lipids, and other non-protein components, that play key roles in plaque/NFT deposition, stability, and clearance. Thus, it is imperative to utilize complementary analytical approaches, such as glycomics, lipidomics, glycoproteomics, phosphoproteomics, and imaging mass spectrometry, to characterize the molecular composition and biochemical environment of plaques and NFTs completely. For example, a recent nanoPhos spatial phosphoproteomics workflow introduced by the Mann group [82] combines laser microdissection with phosphoproteomics in brain tissue, extending their deep visual proteomics method [41] to include PTMs.

While global proteomic analyses have been successfully applied to AD lesions, PTM-specific studies remain sparse, particularly those focused on phosphorylation and glycosylation within individual plaques or NFTs [69,83]. Most available datasets are derived from bulk tissue extracts or sarkosyl-insoluble fractions, limiting spatial resolution and lesion specificity [63,84,85,86]. Intact glycopeptide profiling with site-level resolution and structural glycan identification is lacking for both plaques and NFTs. Only a few tau glycosylation sites—most notably O-GlcNAc modifications—have been characterized by mass spectrometry (MS) in AD brain tissue [87]. Additionally, although the crosstalk between PTMs (e.g., O-GlcNAcylation antagonizing tau phosphorylation) is biologically relevant, very few MS studies investigate multiple PTMs on the same peptide or in a lesion-resolved context [88]. Immunohistochemical and lectin-based imaging studies have shown selective enrichment of α-2,6-linked N-sialic acids in the microglial environment surrounding Aβ plaques in human and mouse models, but not within the plaques themselves, further illustrating the spatial complexity of glycosylation patterns in AD pathology [89].

Several technical challenges impede progress in this area. Aggregated protein assemblies in plaques and NFTs are heavily crosslinked, rendering them resistant to proteolytic digestion and conventional PTM enrichment techniques, such as immobilized metal affinity chromatography (IMAC) for phosphopeptides and hydrophilic interaction chromatography (HILIC) for glycopeptides [16]. Moreover, PTM analyses typically focus on detergent-soluble fractions, thereby excluding probably the most disease-relevant insoluble aggregates [90]. Even large-scale phosphoproteomics studies that utilize bulk tissues (e.g., label-free and TMT-based) often yield qualitative or semi-quantitative PTM data, lacking lesion-specific information [91]. Temporal dynamics of PTMs also remain understudied; most phosphorylation data derive from end-stage AD tissue, while glycosylation remains largely unmapped across disease stages and brain regions. Glycoproteomic data specific to amyloid plaques are similarly limited, with few reports derived from glyco-enriched or lesion-enriched fractions.

To address these gaps, future studies should prioritize LMD-based phospho- and glycoproteomic workflows (e.g., LMDTMT and LMD-DIA-MS) targeting individual plaques and tangles. Developing robust pipelines for intact glycopeptide analysis with structure-sensitive fragmentation would enable detailed mapping of lesion-associated glycosylation. Likewise, the implementation of dual PTM enrichment strategies could elucidate interactions between modifications, such as O-GlcNAcylation and phosphorylation. Improved extraction and digestion protocols for heavily aggregated, insoluble proteins are also critical. Finally, applying quantitative mass spectrometry approaches—such as SILAC, TMT, and DIA-MS—across disease stages and brain regions will help map dynamic PTM patterns and identify potential biomarkers and therapeutic targets.

By addressing these technological and methodological shortcomings, the field can move closer to a comprehensive, molecularly resolved understanding of how PTMs regulate AD lesion biology, facilitating the discovery of novel diagnostic and therapeutic strategies.

## 5. Conclusions

Proteomic profiling of amyloid plaques and neurofibrillary tangles has provided critical insights into their molecular composition, identifying both established and novel proteins. Methodological challenges in sample preparation, spatial resolution, and PTM detection limit our ability to fully capture the complexity of these lesions. Furthermore, differences in analytical approaches, such as LMD versus bulk extraction, yield complementary but sometimes divergent profiles, underscoring the need for integrated strategies that balance molecular depth with anatomical precision. Consideration of patient-specific factors, such as ApoE genotype or PSEN1 mutation status, further underscores the importance of incorporating genetic and clinical context into proteomic analyses. Importantly, the detection of proteins and PTMs within plaques or tangles alone does not establish pathogenic relevance; rigorous functional assays, longitudinal sampling, and independent cohort validation are essential to identify causal drivers of disease. Advances in glycoproteomics, phosphoproteomics, imaging mass spectrometry, and optimized extraction protocols are beginning to expand lesion-specific coverage of key PTMs, particularly phosphorylation and glycosylation. Moving forward, standardized sample handling, PMI-matched controls, and integration with multi-omics and imaging approaches will be critical to achieving a comprehensive molecular map of AD lesions. Such efforts will enable the identification of robust biomarkers and provide mechanistic insights to guide the development of targeted therapeutic strategies.

## Figures and Tables

**Table 2:** Comparison of methodologies for characterizing neurofibrillary tangles (NFTs) in Alzheimer’s disease (AD). This table summarizes four approaches for extracting and analyzing NFTs in AD. Techniques include fluorescence-activated cell sorting (FACS) from post-mortem brain tissue [53]; single-step sarkosyl-based extraction; non-denaturing sarkosyl-based extraction followed by sucrose gradient centrifugation [54]; in vivo positron emission tomography (PET) imaging using radiotracer ^18^F-MK-6240 [55]; cell-based modeling; and cryo-EM [56]. Table highlights differences in invasiveness, structural preservation, sensitivity, throughput, and potential for downstream applications.

Method	Material	Advantages	Limitations	Reference
Fluorescence-activated cell sorting (FACS). Cells labeled with antibodies (AT8, T22, PHF1).	Postmortem human AD brains.	Non-denaturing. High purity enrichment. Single-cell resolution. Downstream analysis.	Plaque’s contamination. Time-consuming. Low yield. Technically demanding.	[53]
Single-step sarkosyl-based extraction from postmortem brain homogenates.	Sarkosyl-insoluble fraction enriched in phosphorylated tau, including AT8/Pt231-positive high molecular weight tau aggregates.	Fast. Simple. Enriches pathological tau. Avoids harsh denaturants.	Low purity. May co-isolate minor soluble proteins.	[54]
PET imaging with a radiotracer.	Injected with ^18^F-MK-6240, which binds to aggregate tau protein in the brain region.	Non-invasive. Highly selective and sensitive. Minimal off-target binding.	Small sample size. Uncertain off-target effects. Atypical binding.	[55]
Cell-based model, sarkosyl enrichment of insoluble tau, followed by cryo-EM analysis.	Seeded undifferentiated SH-SY5Y neuroblastoma cells expressing human 1N3R or 1N4R tau with brain-derived extracts from AD or corticobasal degeneration (CBD) patients.	Structural insights. Controlled cell-based model. High-resolution structural determination.	Cultured cells may not fully represent in vivo modifications. High cost and technical complexity. Complex workflows and data processing.	[56]

**Table 3:** Comparative overview of recent mass spectrometry-based proteomic studies analyzing amyloid plaques in Alzheimer’s disease. Xiong et al. [39] performed large-scale cross-species proteomic comparison of amyloid plaques containing regions, which were microdissected using laser microdissection (LMD), identifying over 4000 proteins. Jiao et al. performed single amyloid plaque data independent acquisition (DIA) analysis and identified ~5000 proteins. Drummond et al. [60] explored shared plaque proteins between AD and Down syndrome, and Levites et al. [61] characterized conserved Aβ responsome in both human and mouse models, identifying Midkine and Pleiotrophin as potential modulators of pathology. Drummond et al. developed method of capturing amyloid plaque-containing regions using LMD on brain FFPE tissues, followed by downstream mass spectrometry proteomics analysis [38].

Method	Samples	Advantages	Limitations	Findings	Reference
Amyloid plaques containing regions from brain sections from patients with AD, controls, and APP/PS1 transgenic mice were collected using LMD.	Amyloid plaque containing regions and Adjacent non-amyloid Plaque containing regions.	Comprehensive analysis: quantifying a large number of proteins and providing a detailed proteome of amyloid plaque-containing regions. Comparative approach: comparing AD plaque-containing regions from non-AD brains and APP/PS1 mice. Identifying potential biomarkers: by providing specific proteins upregulated in AD plaque-containing regions: suggests potential biomarkers for early detection and therapeutic targeting.	Sample Size: The study’s findings are based on a limited number of samples, which may affect the generalizability of the results. Tissue Heterogeneity: Amyloid plaques are heterogeneous, and the study’s approach may not capture all variations within different plaque types. Lack of Functional Validation: While the study identifies proteins enriched in amyloid plaque-containing regions, it does not provide functional validation of their roles in AD pathology.	Over 4000 proteins quantified across amyloid plaque-containing regions. At least 40 proteins were identified as highly enriched in AD and non-AD brains, including APCS, ApoE, midkine, VGFR1, and complement C4. In AD brains, the amyloid plaque-containing regions included synaptic structural proteins and complement C1r, C5, and C9.	[39]
Capturing single amyloid plaques containing regions from fresh frozen brain tissue for subsequent profiling using DIA proteomics. Fresh frozen tissue sections were fixed with 70% alcohol and stained for plaques using X-34 dye with serial dehydration. One plaque and one non-plaque containing area from the same section were captured using LMD (approximately 50 µm diameter). Due to the low protein levels in plaque-containing regions (~5 ng/plaque), 0.2% DCA was used as a lysis buffer. This buffer can precipitate upon acidification, eliminating the need for a desalting step and minimizing sample loss.	Tissue sections (10–12 µm) were collected from fresh frozen mouse and human brains.	Spatial precision: precise capturing of individual plaque-containing regions from fresh-frozen brain tissue, facilitating in-depth proteomic profiling at the sub-microgram level. High sensitivity and Specificity-The combination of LMD and DIA mass spectrometry allows for detailed proteomic analysis of amyloid plaque-containing regions, providing insights into the molecular composition of these structures. Minimum protein loss, as it does not require additional steps like desalting.	Low Sample size. Labor-intensive. Time-consuming. Requires specialized equipment and expertise, which may limit its widespread application. Loss of spatial proteoforms.	~20,000 peptides and ~5000 proteins have been identified from ~5 ng initial protein per sample. Several key proteins, such as Abeta, Apo, Mdk, and Ntn1, consistently appeared in the plaque-containing areas.	[62]
Formalin-fixed paraffin-embedded tissues stained with Aβ antibodies. Analyzed amyloid plaques containing regions of AD and Down Syndrome using LC-MS/MS.	Amyloid plaque regions and adjacent non-amyloid plaque regions. 48 proteins consistently enriched in amyloid plaques containing regions across AD and Down syndrome.	Subtyped comparison comparing early onset AD and Down syndrome with AD, providing insights into proteomics differences in different diseases. Targeted enrichment of amyloid plaques using LMD enhanced proteome specificity.	Samples derived from FFPE are subject to fixation artifacts, crosslinking, and extraction biases, potentially impacting protein recovery and detection. The study lacks functional validation of proteome findings.	Observed 48 proteins that were frequently in plaque-containing regions. MDK, COL25A1, SMOC1, NTN1, OLFML3, HTRA1, and APCS proteins were consistently enriched in amyloid plaque-containing regions. Noticed endosomal/lysosomal proteins in high concentrations. As well as phosphorylated Aβ, pyroglutamate Aβ, and Aβ oligomers.	[60]
Comparative LC-MS/MS analysis of brain proteomes from Alzheimer’s disease (AD) patients and Aβ-depositing mouse models (e.g., 5x FAD, APP-KI).	Protein network (M42) enriched in amyloid plaques containing regions, cerebrovascular amyloid (CAA), and dystrophic neurites across AD and mouse models.	Cross-species proteomic integration: integrated and compared human AD proteomes with those from Aβ-depositing mouse models and identified a conserved set of proteins—the “Aβ amyloid responsome”—that persistently associate with amyloid pathology. Functional validation of two key proteins, MDK and CAA, suggests these proteins are active pathology modifiers.	Complexity of protein networks. Lack of spatial resolution: the study relies on bulk proteomic and network analyses rather than spatial methods, limiting the understanding of plaque microenvironments and regional heterogeneity.	Identified a conserved group of proteins—module M42. M42 proteins co-localized in amyloid plaque-enriched regions, dystrophic neuronal processes, and cerebral amyloid angiopathy. Overexpression of MDK and PTN promoted deposition of Aβ in plaques and CAA. M42 directly binds to Aβ fibrils.	[61]
Microdissection of plaque-containing regions and NFTs from archived AD tissue using LMD followed by LC-MS/MS analysis.	FFPE human tissue samples.	Spatial enrichment of amyloid plaques. Preserve tissue architecture. Detect a broad range of proteins and perform unbiased profiling.	Labor-intensive workflow. Limited quantitative precision. Tissue heterogeneity may complicate data interpretation.	Microdissecting 2 mm^2^ of plaques takes 2 h and analyzes about 900 proteins in downstream LC-MS/MS analysis.	[38]

**Table 4:** Comparative overview of recent mass spectrometry-based proteomic studies analyzing neurofibrillary tangles (NFTs) in Alzheimer’s disease (AD). This table outlines methods used for enriching and analyzing neurofibrillary tangles (NFTs) or tau-associated proteins in AD. Approaches include cerebrospinal fluid (CSF) profiling using PET imaging tracers [68]; determining sarkosyl insoluble tau from various tauopathies by LC-MS/MS PTM analysis [69]; method of enriching NFT using laser microdissection (LMD) on brain FFPE tissues, followed by downstream mass spectrometry proteomics analysis [38]; detergent insoluble proteome analysis using tandem mass tag corrected (TMTc) quantitative mass spectrometry [70]; and two dual method proteomic approach with LMD mass spectrometry (LMD-MS) and affinity purification MS [71]; determining immunoprecipitated sarkosyl soluble and insoluble tau fractions by high-resolution MS with isotope-labeled standards [72]; and quantitative proteomics of tau and Aβ in detergent fractions from AD brains [73]. Each method is evaluated based on method, enriched material, and findings. These strategies differ in invasiveness, protein yield, sensitivity, and capacity for direct NFT analysis, providing complementary insights into tau-related disease mechanisms.

Method	Samples	Advantages	Limitations	Findings	Reference
Profiling of cerebrospinal fluid using the O-link Explore 3072 panel and correlating protein levels with in vivo tau tangle burden measured by tau PET imaging.	Cerebrospinal fluid labelled with RO948 tracer.	Detailed molecular profiles. Scalability. Integration of in vivo imaging with proteomics. Identification of stage-specific protein signatures.	Lack of direct NFT enrichment, which may affect specificity for tangle-associated processes. Reliance on CSF rather than direct brain tissue may not fully capture region-specific or plaque-localized pathology present in brain tissue.	Identified 127 differentially abundant proteins. Proteins correlated with tau PET burden and accumulated in neuronal origins related to synaptic transmission, ATP metabolism, and mitochondrial function. Observed that increased accumulation of tau grouped into a co-expression module enriched for neuronal activity and energy metabolism. Proteins that were associated the most with tau PET: MAPT, FABP3, MIF, NRGN, ENO1/2, GLODH.	[68]
Sarkosyl insoluble tau from various tauopathies linked to chromosome 17 with tau inclusions, followed by LC-MS/MS analysis.	Brains of patients with Alzheimer’s disease, Pick’s disease, progressive supranuclear palsy, corticobasal degeneration, globular glial tauopathy, and frontotemporal dementia, and Parkinsonism.	Broad disease coverage: study included multiple tauopathies. Use of multiple brain regions reduces the region-specific bias. Comprehensive PTM mapping, including phosphorylation, acetylation, and ubiquitination. Validation of MS PTM findings using immunoblotting.	Postmortem interval (PMI) and protein degradation could influence PTM detection. Lack of functional assays. Focus on insoluble tau fractions, thus missing PTMs on soluble tau species that could be relevant for early pathology.	170 PTMs in total were identified, including new PTMs. The PTMs included phosphorylation sites focused in the 181–238 and 396–422 regions of the tau, corresponding N- and C-terminal flanking regions of the microtubule binding repeats. Other reported PTMs include ubiquitination and deamidation.	[69]
Microdissection of plaque-containing regions and neurofibrillary tangles from archived AD tissue.	FFPE human tissue samples.	Spatial enrichment of amyloid NFTs. Preserve tissue architecture. Detect a broad range of proteins and perform unbiased profiling.	Labor-intensive workflow. Limited quantitative precision. Tissue heterogeneity may complicate data interpretation.	Microdissecting 1.5 mm^2^ of NFTs takes 8 h and identifies about 500 proteins in downstream LC-MS/MS analysis.	[38]
Detergent insoluble proteome in AD using tandem mass tag corrected (TMTc) quantitative mass spectrometry.	Human postmortem brain tissue samples (frontal gyrus) from human AD and control patients.	Use of TMTc-corrected quantitation improved quantitative accuracy and reduced ratio distortion common in isobaric labeling. High proteome coverage—identified and quantified a broad range of insoluble proteins, including amyloid-associated, cytoskeletal, and synaptic proteins.	Loss of soluble proteins. Lacks spatial and localization data (e.g., whether proteins are within plaques, tangles, or other aggregates).	Meta-analysis of two independent detergent-insoluble AD proteome datasets (8914 and 8917 proteins) was performed. 190 differentially expressed proteins in AD vs. control. Altered pathways included amyloid cascade (amyloid beta binding, amyloid fibril formation), RNA splicing, extracellular matrix, endocytosis/exocytosis, protein degradation, and synaptic activity pathways. Using enrichment factor analysis to distinguish aggregated proteins from copurified components, 84 upregulated proteins among differentially expressed proteins were in the enriched list, suggesting they belong to aggregating or co-aggregating proteins in AD. 84 proteins harbor low complexity regions in their sequences, including amyloid-β, tau, TARDBP/TAR DNA-binding protein 43, SNRNP70/U1-70K, MDK, PTN, NTN1, NTN3, and SMOC1.	[70]
LMD—MS.	NFT-containing neurons from post-mortem human brain tissue.	Use of immunoaffinity purification + MS enabled specific capture of tau-associated protein complexes from human brain tissue. Focused on phosphorylated tau (p-tau) interactome. Comprehensive interactome mapping—identified numerous tau-binding partners, including cytoskeletal, synaptic, RNA-binding, and mitochondrial proteins, highlighting tau’s broad cellular impact.	Antibody enrichment may cause potential bias by capturing proteins associated with the specific phosphorylated tau epitopes and may miss interactions with other tau conformations. Lack of spatial resolution—does not indicate whether identified interactors co-localize with tau in specific brain regions or cell types.	Identified 542 proteins found in NFTs; commonly known proteins associated with NFTs: tau protein, ubiquitin, neurofilament proteins, and ApoE.	[71]
Affinity Purification MS.	Proteins interacting with phosphorylated tau (using PHF1 antibody).			Confirmed 75 proteins interact with PHF1-immunoreactive p-tau. Linked 34 new proteins to p-tau. E.g., VAMP2, NSF, PURA. Discovered 12 novel proteins that have not previously been known to be physiologically or pathologically associated with tau, e.g., HNRPA1.	
Soluble (tris-buffered saline, TBS) and sarkosyl-insoluble (SI) fractions were immunoprecipitated using antibodies targeting all four tau regions. Tryptic peptides corresponding to isoforms and peptides carrying one, two, or three phosphorylations were subjected to LC-MS/MS analysis using isotope-labelled protein and phospho-peptide standards for quantification.	Frontal cortices from AD (*n* = 10), progressive supranuclear palsy (PSP, *n* = 11), Pick’s disease (PiD, *n* = 10), corticobasal degeneration (CBD, *n* = 10), and controls (*n* = 10).	Comparison across tauopathies, including AD, PSP, CBD, and other tauopathies, identified shared vs. disease-specific tau PTM patterns. Region-specific analysis of tau isoform distribution and phosphorylation across brain areas eliminated region-specific bias.	While region-level differences were measured, subcellular or lesion-specific localization was not addressed.	0N and 1N tau isoforms were most abundant. Increase in the 0N isoform, and double and triple-phosphopeptides in the SI fraction in AD. SI fraction also showed the 3R/4R isoform predominance characteristic of the different tauopathies, with the 3R being more abundant in PiD, 4R in PSP and CBD, while they had similar abundances in AD and controls. Microtubule-binding region (MTBR) significantly more abundant in AD, indicating aggregation.	[72]
Quantitative proteomics of tau and Aβ in detergent fractions from AD brains. Sarkosyl-soluble and -insoluble extracts to characterize tau and Aβ species by quantitative mass spectrometric proteomics, biochemical assays, and electron microscopy.	Age-matched AD brains (*n* = 11) and disease-control Amyotrophic Lateral Sclerosis (ALS) brains (*n* = 10).	Fractionation of brain homogenates into detergent-soluble and detergent-insoluble fractions enabled MS analysis of aggregated, pathology-associated protein species. Parallel assessment of tau and Aβ proteomes.	Lacks spatial context and functional validation.	AD brain sarkosyl-insoluble pellets were greatly enriched with Aβ42 at almost equimolar levels to N-terminal truncated MTBR isoforms of tau with multiple site-specific PTMs. MTBR R3 and R4 tau peptides: most abundant in the sarkosyl-insoluble fraction with a 10-fold higher concentration than N-terminal tau peptides. High concentration and occupancies of site-specific phosphorylation pT181 (~22%) and pT217 (~16%).	[73]

## Data Availability

No new data were generated from this article.

## Abbreviations

Alzheimer’s disease, AD; neurofibrillary tangles, NFTs; mass spectrometry, MS; matrix-assisted laser desorption ionization, MALDI; mass spectrometry imaging, MSI; laser microdissection, LMD; immunohistochemistry, IHC; liquid chromatography–tandem mass spectrometry, LC-MS/MS; post-translational modifications, PTMs; formalin-fixed paraffin-embedded; FFPE.
